# A correlative analysis of epidemiologic and molecular characteristics of methicillin-resistant *Staphylococcus aureus* clones from diverse geographic locations with virulence measured by a *Caenorhabditis elegans* host model

**DOI:** 10.1007/s10096-012-1711-x

**Published:** 2012-08-18

**Authors:** K. Wu, K. Zhang, J. McClure, J. Zhang, J. Schrenzel, P. Francois, S. Harbarth, J. Conly

**Affiliations:** 1 Centre for Antimicrobial Resistance, Alberta Health Services/Calgary Laboratory Services/University of Calgary, Calgary, AB Canada; 2 Department of Microbiology, Immunology and Infectious Diseases, University of Calgary, Calgary, AB Canada; 3 Department of Pathology and Laboratory Medicine, University of Calgary, Calgary, AB Canada; 4 Department of Medicine, University of Calgary, Calgary, AB Canada; 5 The Calvin, Phoebe and Joan Snyder Institute for Chronic Diseases, University of Calgary, Calgary, AB Canada; 6 Genomic Research Laboratory, University of Geneva Hospitals and Faculty of Medicine, Geneva, Switzerland; 7 Infection Control Program, University of Geneva Hospitals and Faculty of Medicine, Geneva, Switzerland; 8 Department of Medicine, Foothills Medical Centre, 1403 29th Street NW, Calgary, AB T2N 2T9 Canada

## Abstract

Methicillin-resistant *Staphylococcus aureus* (MRSA) strains from different geographic areas have different genetic backgrounds, suggesting independent clonal evolutions. To better understand the virulence of MRSA strains and the relationship to their clonal and geographic origins, we undertook an analysis of epidemiologic, molecular, and virulence characteristics of a large number of MRSA isolates from geographically diverse origins, in a *Caenorhabditis elegans* infection model. A total of 99 MRSA isolates collected between 1993 and 2010 at the Geneva University Hospitals from diverse global origins were characterized with Panton–Valentine leukocidin (PVL), toxic shock syndrome toxin (TSST), accessory gene regulator (*agr*) group, staphylococcal cassette chromosome *mec* (SCC*mec*), *S. aureus* protein A (*spa*), multilocus sequence typing (MLST), and pulsed-field gel electrophoresis (PFGE) typing. Epidemiologic data were provided from clinical records. The bacterial virulence was tested in a *C. elegans* host model. The inter-relationships of epidemiological/molecular characteristics in association with nematocidal activities were analyzed with univariate and two-factor analysis of variance (ANOVA). Community-associated MRSA (CA-MRSA) strains were more virulent than hospital-associated MRSA (HA-MRSA), with higher nematocidal activities in CA-MRSA strains (0.776 vs. 0.506, *p* = 0.0005). All molecular characteristics (PVL, TSST, *spa*, SCC*mec*, MLST, and PFGE types) showed a significant association with nematocidal activities on univariate analysis (*p* < 0.005). PVL was not a significant predictor after adjusting for genomic backgrounds using *spa*, MLST, or PFGE typing. The dominant CA-MRSA strains in North America showed higher nematocidal activities than strains from other regions (*p* < 0.0001). Strains with global origins containing distinct genetic backgrounds have different virulence in the *C. elegans* model. Nematocidal activities were most highly correlated with SCC*mec*, *spa*, MLST, and PFGE typing, suggesting that genomic background rather than a single exotoxin characteristic was the most discriminating predictor of virulence.

## Introduction

Methicillin-resistant *Staphylococcus aureus* (MRSA) infections have been reported in the hospital and community settings worldwide since the first case was identified in the United Kingdom [1]. With the evolving epidemiology and development in molecular typing methods for *S. aureus*, it has become possible to study the population and evolutionary biology of MRSA on a larger geographic level. Based on multilocus sequence typing (MLST), there are currently 17 major clonal complexes (CCs) identified from the *S. aureus* isolates collected worldwide, including methicillin-susceptible *S. aureus* (MSSA) and MRSA strains [2]. For hospital-associated MRSA (HA-MRSA), the Iberian (ST247), Brazilian (ST239), Paediatric (ST5), EMRSA15 (ST22), EMRSA16 (ST36), and Berlin (ST45) clones are recognized pandemic clones in the world [3]. However, community-associated MRSA (CA-MRSA) have different patterns, with the major CA-MRSA clones being ST1, ST8, ST30, ST59, ST80, and ST88, plus other minor clones, circulating around the world [4–6]. ST1 and ST8 CA-MRSA, also named as USA400 and USA300, respectively, are two dominant CA-MRSA strains in North America. ST30, ST59, ST80, and ST88 are successful CA-MRSA strains present in Australia, Taiwan, Europe, and Africa, respectively [7, 8].

The reason for the distinct epidemiologic patterns of MRSA clones in different geographic areas is unknown. Previous studies have shown that strain ST8 has enhanced virulence in human infection and animal models, which may contribute to its dominance in North America [9–12]. However, whether there are differences in virulence between the strains circulating in the community in different continents, such as ST8, ST80, or ST30 strains, has not been fully investigated.

We have previously shown that CA-MRSA is more virulent than HA-MRSA using an invertebrate *Caenorhabditis elegans* host model, correlating the findings with human clinical data [13]. In an effort to better understand the virulence of MRSA strains and their relationship to their clonal and geographic origins, we analyzed the bacterial virulence of a large number of MRSA isolates from geographically diverse origins using a *C. elegans* infection model, and undertook a detailed epidemiologic, molecular, and virulence correlative analysis of these isolates.

## Materials and methods

### Bacterial strains and isolates

A total of 99 isolates, during a 17-year period (1993–2010), were obtained from retrospective specimen collections , the details of which are described elsewhere [7, 14], in the Geneva University Hospitals, a 2,200-bed primary and tertiary medical center in Switzerland These isolates were obtained from the original stocks which had been retained in the freezer over the years. The isolates were separated into different categories based on their clinical sites, including colonization, skin and soft tissue infection (SSTI), pulmonary infection, mastitis, urinary tract infection, otitis externa, septic arthritis, and bloodstream infection. These isolates were also separated into hospital-associated and community-associated strains based on the presence of the infection or colonization within 48 h after hospital admission. Reference strains CMRSA1–10 and USA100–1000 were provided by the National Microbiology Laboratory (NML), Health Canada (Winnipeg, Manitoba, Canada), and by the Network on Antimicrobial Resistance in *Staphylococcus aureus* (NARSA), respectively.

### Molecular and genomic characterization of isolates

Genomic DNA isolated from a single colony was tested by multiplex real-time polymerase chain reaction (PCR) for staphylococcal cassette chromosome *mec* (SCC*mec*) elements, accessory gene regulator (*agr*) group, Panton–Valentine leukocidin (PVL), and toxic shock syndrome toxin-1 (TSST) [7]. The presence of arginine deiminase (*arc*A) was assessed by PCR-based assays (*arc*A-F: GCAGCAGAATCTATTACTGAGCC; *arc*A-R: TGCTAACTTTTCTATTGCTTGAGC). MLST typing and pulsed-field gel electrophoresis (PFGE) were performed as previously reported [15, 16]. PFGE clusters were defined according to the criteria described by Tenover et al. [17].

### *C. elegans* survival assay

The virulence of all 98 isolates (one isolate forming a thick bacterial lawn was excluded) was tested in triplicate, using a *C. elegans* host model, with the strains NCTC8325 and M92 representing positive and negative reference strains, respectively [13]. Briefly, Bristol N2 *C. elegans* nematodes were maintained at room temperature (RT) on nematode growth medium (NGM) plates seeded with *Escherichia coli* strain OP50 as a food source. A 10-μl aliquot of 10× diluted overnight culture of *S. aureus* in brain–heart infusion (BHI) broth was spread into 3.5-cm-diameter plates containing tryptic soy agar (TSA) supplemented with 5 μg/ml nalidixic acid (NA) and incubated at 37 °C for 4–6 h. Thirty 4th larval (L4) stage hermaphrodite nematodes were transferred from *E. coli* OP50 NGM plates to the assay TSA plates grown with the tested isolates, and the plates were kept at RT. Their survival was monitored every 24 h over a 5-day period. Data were analyzed by the Kaplan–Meier method for nematode survival rate using GraphPad Prism (GraphPad Software, La Jolla, CA, USA). To compare the nematocidal activities from each individual experiment, all the nematocidal activities were calibrated with the positive reference strain (nematocidal activity referenced as 1) and the negative reference strain (nematocidal activity referenced as 0). The calibrated death rate, representing the mean of the triplicate testing, was calculated as Δdeath rate (test strain-M92)/Δdeath rate (NCTC8325-M92).

### Statistical methods

Student’s *t*-test and the single-factor analysis of variance (ANOVA) test were used to determine whether the epidemiological or molecular characteristics, including community/hospital association, *pvl*, *tsst-1*, *agr*, ST, PFGE, or *spa* types, were associated with bacterial virulence based on the calibrated nematocidal activity. Two-factor ANOVA testing (SPSS v15, IBM, USA) was used to investigate the inter-relationships between characteristics found to be significant in the univariate analysis using a level of significance of 0.05. Isolates were excluded from the analysis if they fell into a group containing less than three isolates.

## Results

### Epidemiology of Geneva isolates

**Fig. 1:**
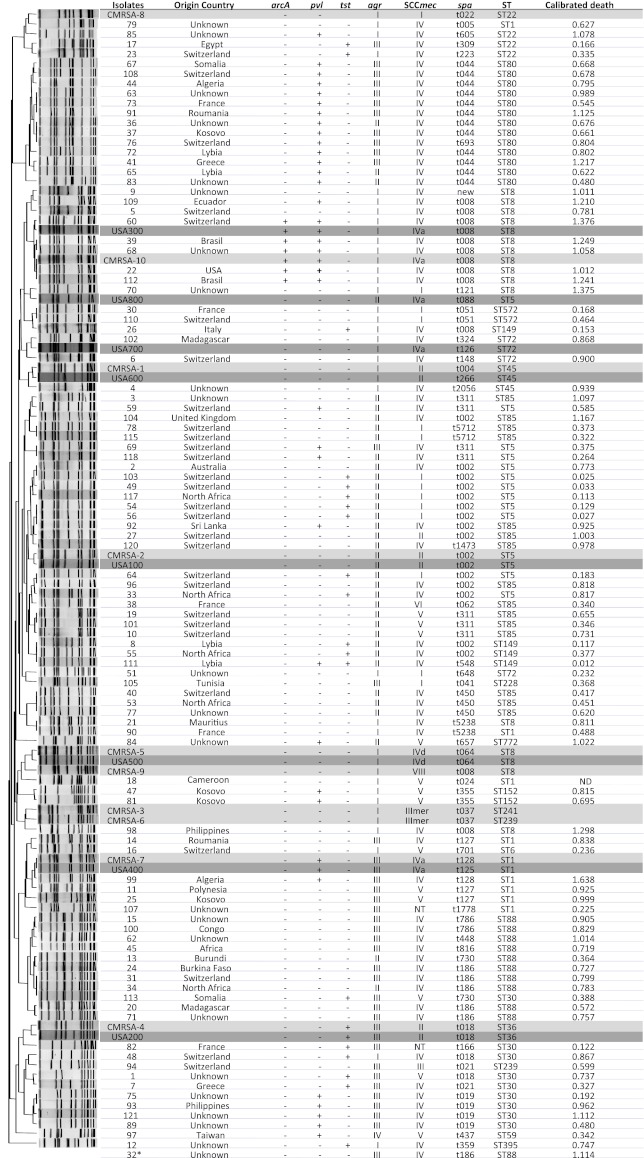
Global origins and molecular features of Geneva isolates. USA100–800 and CMRSA1–10 were used as reference strains, highlighted in gray and dark gray, respectively. *arc*A, arginine deiminase of arginine catabolic mobile element (ACME) from USA300; PVL, Panton–Valentine leukocidin; TSST, toxic shock syndrome toxin; *agr*, accessory gene regulator; SCC*mec*, staphylococcal cassette chromosomal *mec*; *spa*, *Staphylococcus aureus* protein A; ST, sequence type; calibrated death, the nematocidal activity of Geneva isolates normalized with positive and negative control strains in the *Caenorhabditis elegans* model. One strain was nontypable by pulsed-field gel electrophoresis (PFGE); two strains were untypable using the available SCC*mec* typing method and one strain (#18) could not be reliably tested in the *C. elegans* model

A total of 99 isolates were collected from 99 patients [mean ± standard deviation [SD] age: 36 ± 25 years] travelling from or living in different continents, including Europe, North America, South America, Africa, Asia, and Australia (Fig. 1). Of the total number of isolates, 40 were hospital-associated, 47 were community-associated, and 12 had unknown origins (Table 1). Almost half of the isolates were identified as colonizing isolates and 36 isolates were associated with SSTI. Nine isolates were associated with other infections, including bloodstream infection, septic arthritis, urinary tract infection, mastitis, otitis externa, and tracheobronchitis. The cases of otitis externa and tracheobronchitis may be considered less invasive than the other infections. The clinical manifestations associated with the remaining nine isolates were unknown. When these isolates were grouped into colonization and infection isolates (Table 1), the relative ratios of colonization versus infection for the community-associated (16/31, 0.52) and the hospital-associated (26/14, 1.86) isolates, respectively, were significantly different from each other (*p* = 0.0064 by the χ^2^ test).

**Table 1:** Epidemiologic profiles of 99 isolates collected in Geneva University Hospitals, 1993–2010, stratified by their origin (community vs. hospital)

Clinical sites		Community	Hospital	Unknown source
Colonization		16	26	3
Infection	SSTI	27	9	
	Tracheobronchitis		2	
	Otitis externa	2		
	Mastitis	1		
	UTI		1	
	Septic arthritis	1		
	Bloodstream infection		2	
No records				9
Total		47	40	12

*SSTI*, skin and soft tissue infection; *UTI*, urinary tract infection

### Molecular characteristics of Geneva isolates

There were 39 *spa*, 19 ST, and 26 PFGE types (one isolate untypable) identified among the 99 isolates (Fig. 1). All isolates were resistant to methicillin, and carried type I, II, III, IV, V, or VI SCC*mec* elements, except for two isolates, both of which contained *mec*A, but were unable to be typed with the available methodology. The PVL gene was only found in SCC*mec* IV and V isolates. Moreover, the majority of these isolates belonged to *agr* I, II, or III, with only one isolate carrying *agr* IV (Fig. 1).

MLST showed that ST1, ST5, ST8, ST30, ST80, ST85, and ST88 were the major ST groups with more than five isolates. ST8-SCC*mec* IV isolates were clustered with the USA300 reference strain, and carried *arc*A, which is located in a unique mobile genetic element (arginine catabolic mobile element, ACME), whereas ST1-SCC*mec* IV isolates were clustered with the USA400 reference strain. All ST1, ST8, ST30, and ST80 carried SCC*mec* IV, except for one ST8 isolate which carried SCC*mec* I and two ST30 isolates which carried SCC*mec* V; in contrast, ST85 and ST5 carried more diversified SCC*mec* elements, such as SCC*mec* I, II, IV, and V.

Both ST8-SCC*mec* IV and ST1-SCC*mec* IV isolates in this collection originated from countries other than North America, such as South America and South East Asia. Strains of ST80 also originated from countries in Northern Africa, in addition to central Europe.

### Correlation between epidemiological/molecular characteristics and nematocidal activities

The virulence of 98 isolates (one isolate could not be reliably tested in the assay) in the *C. elegans* host model was shown as the calibrated death (CD) (Fig. 1). To determine which epidemiological or molecular characteristics were associated with the nematocidal activity in the *C. elegans* model, different comparisons were made among groups carrying the same characteristic.

**Fig. 2:**
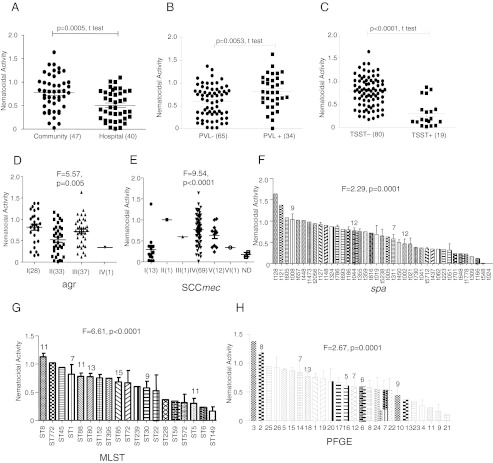
Correlation between different epidemiologic/molecular characteristics and nematocidal activities. The nematocidal activity correlated with different epidemiologic/molecular characteristics: **a** community vs. hospital; **b** *pvl*; **c** *tsst-1*; **d** *agr* type; **e** SCC*mec* types; **f** *spa* type; **g** ST type; **h** PFGE cluster. The numbers in brackets or on top of bars indicate the number of isolates analyzed in the group

There was no significant difference in the mean nematocidal activity in those isolates associated with clinical infection versus those associated with colonization. However, isolates which originated from the community showed significantly higher nematocidal activities than isolates from the hospital (mean CD: 0.776 vs. 0.506, *p* = 0.0005, Fig. 2a). This result correlated with the clinical outcomes, with more isolates from the community causing infections than those from the hospital (Table 1). The comparison between *pvl*+ and *pvl*− isolates showed that *pvl*+ isolates had higher nematocidal activities than *pvl*− isolates (mean CD: 0.815 vs. 0.601, *p* = 0.0053, Fig. 2b). Unexpectedly, the comparison between *tsst-1*+ and *tsst-1*− isolates showed that *tsst-1*− isolates had significantly higher nematocidal activities than *tsst-1*+ isolates (mean CD: 0.766 vs. 0.299, *p* < 0.0001, Fig. 2c).

Single-factor ANOVA was used to determine whether different *agr*, SCC*mec*, *spa*, ST, or PFGE types were associated with nematocidal activities. For *agr* types, the mean CD of nematocidal activities of *agr* I was 0.816, *agr* II 0.524, and *agr* III 0.717 (F = 5.57, *p* = 0.005; Fig. 2d), respectively. For SCC*mec* types, SCC*mec* IV showed higher virulence than the other SCC*mec* types (F = 9.54, *p* < 0.0001; Fig. 2e). Similarly, the *spa* type t008 showed a greater mean CD than the other groups, excluding the groups with less than two isolates (F = 2.29, *p* = 0.013; Fig. 2f). Moreover, CA-MRSA strains ST8 and ST1 were significantly more virulent than prevalent strains in other geographic areas, including ST88, ST80, ST85, ST30, and ST5, with the mean CD of ST8 isolates, 1.13, being the greatest (F = 6.61, *p* < 0.0001; Fig. 2g). Furthermore, the PFGE cluster 2, corresponding to ST8 and *spa* t008 isolates, had a greater mean CD than the other clusters (F = 2.67, *p* = 0.001; Fig. 2h).

**Fig. 3:**
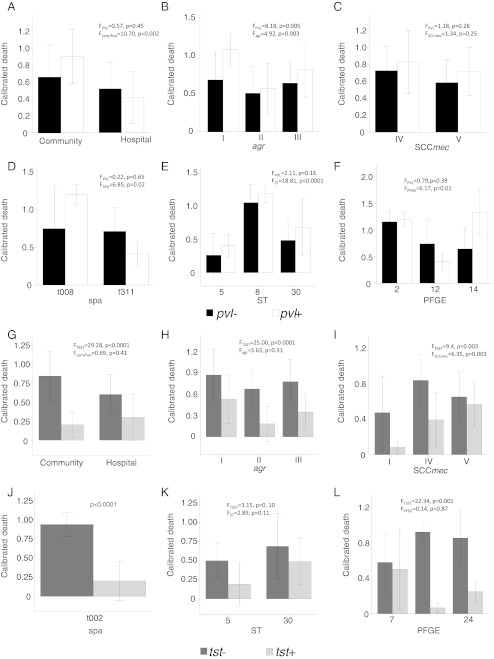
Two-factor analysis of variance (ANOVA) to determine the inter-relationship of PVL or TSST with other epidemiologic/molecular characteristics in association with nematocidal activities. **a–f** The impact of PVL on nematocidal activities is dependent on: **a** community vs. hospital; **b** *agr* type; **c** SCC*mec* type; **d** *spa* type; **e** ST type; and **f** PFGE cluster. **g–l** The impact of TSST on nematocidal activities is independent of: **g** community vs. hospital; **h** *agr* type; **i** SCC*mec* type; **j** *spa* type; **k** ST type; and **l** PFGE cluster

Two-factor ANOVA was employed to investigate the inter-relationship of these epidemiological or molecular factors to determine which factor was an independent factor for predicting bacterial virulence. As shown in Fig. 3a–f, when isolates were divided into groups of community/hospital or different *agr*, SCC*mec*, *spa*, ST, and PFGE types, the nematocidal activities of *pvl*+ and *pvl*− isolates inside each group were not significantly different from each other, except for *agr* types. On the other hand, TSST showed a negative correlation with the nematocidal activities and two-factor ANOVA showed that this impact was independent from other factors, except the ST type (Fig. 3g–l).

Moreover, as shown in Table 2, the factor community/hospital appeared to interact with other molecular factors, including *agr*, *spa*, and PFGE (F < 2.357, *p* = 0.07); and *agr* was a dependent factor related to SCC*mec*, ST, PFGE, and *spa* types (F < 1.346, *p* = 0.265). However, SCC*mec*, ST, PFGE, and *spa* types were independent factors more directly associated with nematocidal activities (F > 1.614, *p* = 0.052), except that ST and PFGE appear to be co-dependent (F < 1.848, *p* = 0.071) (Table 2).

**Table 2:** Two-factor analysis of variance (ANOVA) to determine the inter-relationship of two epidemiological/molecular characteristics in association with nematocidal activities. F-tests and *p*-values of Factor 2 interacting with Factor 1 are listed in the table. Com/hosp, community-/hospital-associated; *agr*, accessory gene regulator; SCC*mec*, staphylococcal cassette chromosome *mec*; *spa*, *Staphylococcus aureus* protein; ST, sequence type; PFGE, pulsed-field gel electrophoresis

Factor2	Factor 1					
	Com/hosp	*agr*	SCC*mec*	*spa*	ST	PFGE
Com/hosp		F = 9.498, *p* < 0.001	F = 1.567, *p* = 0.215	F = 1.172, *p* = 0.286	F = 5.735, *p* = 0.020	F = 1.145, *p* = 0.291
*agr*	F = 2.357, *p* = 0.077		F = 1.346, *p* = 0.265	F = 0.044, *p* = 0.957	F = 0.342, *p* = 0.712	F = 0.378, *p* = 0.687
SCC*mec*	F = 3.051, *p* = 0.007	F = 3.157, *p* = 0.005		F = 5.433, *p* = 0.001	F = 3.524, *p* = 0.004	F = 3.846, *p* = 0.003
*spa*	F = 1.555, *p* = 0.173	F = 1.614, *p* = 0.052	F = 2.151, *p* = 0.006		F = 2.408, *p* = 0.003	F = 1.960, *p* = 0.025
ST	F = 5.237, *p* < 0.001	F = 3.207, *p* < 0.001	F = 4.338, *p* < 0.001	F = 6.607, *p* < 0.001		F = 1.848, *p* = 0.071
PFGE	F = 1.233, *p* = 0.304	F = 2.177, *p* = 0.007	F = 3.369, *p* < 0.001	F = 2.930, *p* = 0.008	F = 1.205, *p* = 0.289	

## Discussion

In this study, we utilized the *C. elegans* host model to investigate the pathogenic mechanisms of different MRSA clones from different geographic regions worldwide. ST8 and ST1 strains, the dominant CA-MRSA strains for North America, showed significantly higher virulence than ST5, ST30, ST80, and ST88 strains, the prevalent CA-MRSA strains in other geographic areas. This result may suggest a competitive advantage for these strains and provides a possible explanation for the unequal dissemination of these strains across North America and the increasing prevalence in some European countries [18, 19]. It is possible that bacterial virulence may be related to fitness in the environment, promoting the enhanced transmissibility of these strains [20]. The ST80 and ST88 strains, showing higher nematocidal activities than the ST5 and ST30 strains, are dominant CA-MRSA strains in central Europe and Africa [6–8, 18, 21]. Recently, DeLeo et al. showed that a historically pandemic MSSA clone, phage-type 80/81, causing infections in hospitals as well as outside of the healthcare setting, was highly virulent in mouse infection models compared with other genetically related clones that were mostly hospital-associated infections, supporting the suggestion that high bacterial virulence contributes to increased transmissibility [22].

However, low bacterial virulence does not necessarily correlate with low prevalence. As shown in our study, the ST30 strain exhibited a relatively low nematocidal activity, but is a dominant CA-MRSA clone in Oceania and the Southwest Pacific [5]. It is possible that CA-MRSA strains originate independently in different geographic areas, and strains with certain genomic features may have become endemic within their originating areas, but may have lower virulence than CA-MRSA strains endemic in other areas. Human or animal travel may have promoted the spread of endemic CA-MRSA strains across continents, with the dominant North American CA-MRSA strain ST8 having been isolated now in Europe and Asia [6, 23–25]. Why ST80 has remained a dominant clone within Europe and Northern Africa, despite the entry of the ST8 strain, is unknown. The presence of ST80 may provide a relative protection from the entry of another strain on a population basis or it may only be a matter of time until the ST8 strain becomes dominant in Europe.

This study also determined which molecular markers would be more reliable predictors for bacterial virulence. Currently, the role of PVL in bacterial pathogenesis is still controversial [26–28]. In the present study, PVL was a dependent factor related to other molecular markers, such as *agr*, SCC*mec*, ST, PFGE, and *spa* types. For example, with the same ST types, *pvl*+ and *pvl*− isolates had similar nematocidal activities, suggesting that PVL alone may not contribute to nematocidal activities. In contrast, the presence of the *tsst-1* gene was associated with less nematocidal activity in the *C. elegans* model, with *tsst-1*+ isolates demonstrating less virulence than *tsst-1*− isolates. TSST is a superantigen stimulating the release of large amounts of proinflammatory factors in human infection, and has been associated with human toxic shock syndrome, which affected menstruating women who were using tampons [29], and it may not be necessary for bacterial virulence in invertebrates that only have innate immunity [30]. Alternatively, the *tsst-1* gene in this study is mostly associated with the isolates with ST5, ST30, and ST149, which have low nematocidal activity (Figs. 1 and 3g). However two-factor ANOVA showed that, with the same ST type, the CDs of *tsst-1*+ and *tsst-1*− isolates were not significantly different, suggesting that the presence of TSST is less correlated with nematocidal activities when the total genomic background is considered (Fig. 3k). The virulence of these isolates appears to be associated with typing methods that correlate with strain differentiation at the genomic level, represented by *spa*, ST, or PFGE types, rather than by toxins produced by a single virulence gene, such as *pvl* or *tsst-1*. Therefore, the molecular markers, *spa*, ST, or PFGE types were the most discriminating predictors of virulence in our *C. elegans* model.

Moreover, the data from the *C. elegans* model and the clinical data were relatively well correlated. Isolates from the community, exhibiting higher nematocidal activities than those from the hospital, were more associated with infection than colonization. These findings are corroborated by a previous study [13] and further validates that the *C. elegans* model is a useful tool to study the virulence of *S. aureus*.

We acknowledge the limitations in our study. We recognize that it is difficult to validate the geographic origins of these organisms, but given the propensity for long-term carriage of MRSA strains, the lack of exogenous cross-transmission of these strains between Swiss citizens resident in Geneva, and previous work that the majority of these strains have not been reported previously in Switzerland [7], we believe that there is evidence supporting origins of the isolates from outside Geneva. Although the 99 isolates in the Geneva University Hospitals collection had diverse worldwide origins, the majority of the isolates originated in Europe or Africa, and there were many countries from which no isolates were collected. However, Geneva is one of the most international cities in the world and, consequently, this unique isolate collection is more diverse than what may have been collected from a single center elsewhere. Moreover, for isolates with certain ST, PFGE, and *spa* types, less than two isolates were available and were excluded in our analysis.

The epidemiology of MRSA is complex and evolving, with multiple factors involved, including bacterial virulence, host immunity, social habits of the host populations, and tremendous variation in local and national MRSA control guidelines. Overall, our study has attempted to provide a new perspective on global CA-MRSA epidemiology by exploring bacterial molecular characteristics and virulence, suggesting that the total genomic background rather than any single factor is the most discriminating factor. This study may also provide insights for MRSA diagnosis and prevention, as some molecular characteristics associated with specific genetic backgrounds are discriminating predictors for bacterial virulence.
